# Rare Ovarian Sex Cord-Stromal Tumor: Diagnostic Pitfalls and Clinical Management

**DOI:** 10.7759/cureus.113249

**Published:** 2026-07-23

**Authors:** Beatriz Sousa Ferreira, Ana Cláudia Rodrigues, Elizabeth Castelo-Branco, Teresa Carvalho, Rita Sousa

**Affiliations:** 1 Gynecology and Obstetrics, Unidade Local de Saúde Trás-os-Montes e Alto Douro, Vila Real, PRT; 2 Gynecologic Oncology, Instituto Português de Oncologia de Coimbra Francisco Gentil, Coimbra, PRT; 3 Medical Oncology, Instituto Português de Oncologia de Coimbra Francisco Gentil, Coimbra, PRT

**Keywords:** aged adult, carboplatin-paclitaxel, diagnostic pitfalls, ovarian neoplasm, sertoli–leydig cell tumor, sex-cord stromal tumor

## Abstract

Poorly differentiated Sertoli-Leydig cell tumors of the ovary are exceptionally rare and often difficult to classify, frequently requiring expert pathology review. Their clinical course can be aggressive, and therapeutic decisions are particularly complex in older patients with comorbidities. We describe a woman in her seventies who presented with advanced disease and underwent extensive cytoreductive surgery, followed by systemic chemotherapy. Despite an initial partial response, she experienced disease progression with recurrent ascites and peritoneal carcinomatosis. This case illustrates the diagnostic pitfalls, the importance of multidisciplinary evaluation, and the therapeutic dilemmas encountered when standard regimens fail. It also underscores the need to balance oncological benefit with quality-of-life considerations in elderly patients with rare ovarian malignancies.

## Introduction

Ovarian sex cord-stromal tumors are a rare group of ovarian neoplasms that arise from the specialized cells responsible for supporting ovarian development and hormone production.

Among these tumors, Sertoli-Leydig cell tumors account for less than 0.5% of primary ovarian neoplasms and most commonly occur in adolescents and young adults, although cases in older women have been described [1-5]. Histologically, these tumors are classified as well differentiated, moderately differentiated, poorly differentiated, or retiform, with poorly differentiated forms being associated with greater diagnostic complexity and more aggressive clinical behavior [3,5,6].

Clinical presentation is variable and may include abdominal distension, pelvic pain, a palpable mass, menstrual irregularities, or endocrine manifestations related to androgen or estrogen production [1,6-8]. However, hormonal symptoms may be absent or subtle, particularly in postmenopausal women and in patients with advanced disease [6-8]. Poorly differentiated tumors may also show overlapping morphological and immunohistochemical features with epithelial, mesenchymal, or undifferentiated neoplasms, allowing them to mimic more common ovarian or uterine malignancies and potentially delaying accurate diagnosis, making diagnosis particularly challenging [3,5].

Because advanced Sertoli-Leydig cell tumors are exceptionally uncommon, evidence to guide treatment is limited. Surgery remains the cornerstone of management, whereas adjuvant chemotherapy is generally considered for advanced-stage, recurrent, or poorly differentiated disease [9,10]. Treatment decisions become particularly difficult in elderly or comorbid patients, in whom the potential benefits of systemic therapy must be balanced against toxicity, functional decline, and quality of life [9-12].

We report a rare case of an advanced and poorly differentiated ovarian Sertoli-Leydig cell tumor in an elderly woman, highlighting the diagnostic pitfalls, the value of hormonal evaluation and expert pathology review, and the therapeutic limitations encountered in aggressive disease.

## Case presentation

A woman in her seventies presented with progressive abdominal distension, early satiety, vomiting, bilateral lower limb edema, and back pain. Imaging demonstrated a large heterogeneous adnexal mass with massive ascites, and laboratory evaluation revealed elevated cancer antigen (CA)-125 and estradiol levels. 

She was referred to a tertiary oncology center with suspected primary gynecologic malignancy. Her medical history included obesity, dyslipidemia, hypertension, and a recently diagnosed pulmonary embolism. She had no previous surgery and was a lifelong non-smoker. Her gynecologic history included one full-term vaginal delivery and natural menopause at 52 years of age. She lived independently, with regular family support.

**Figure 1:**
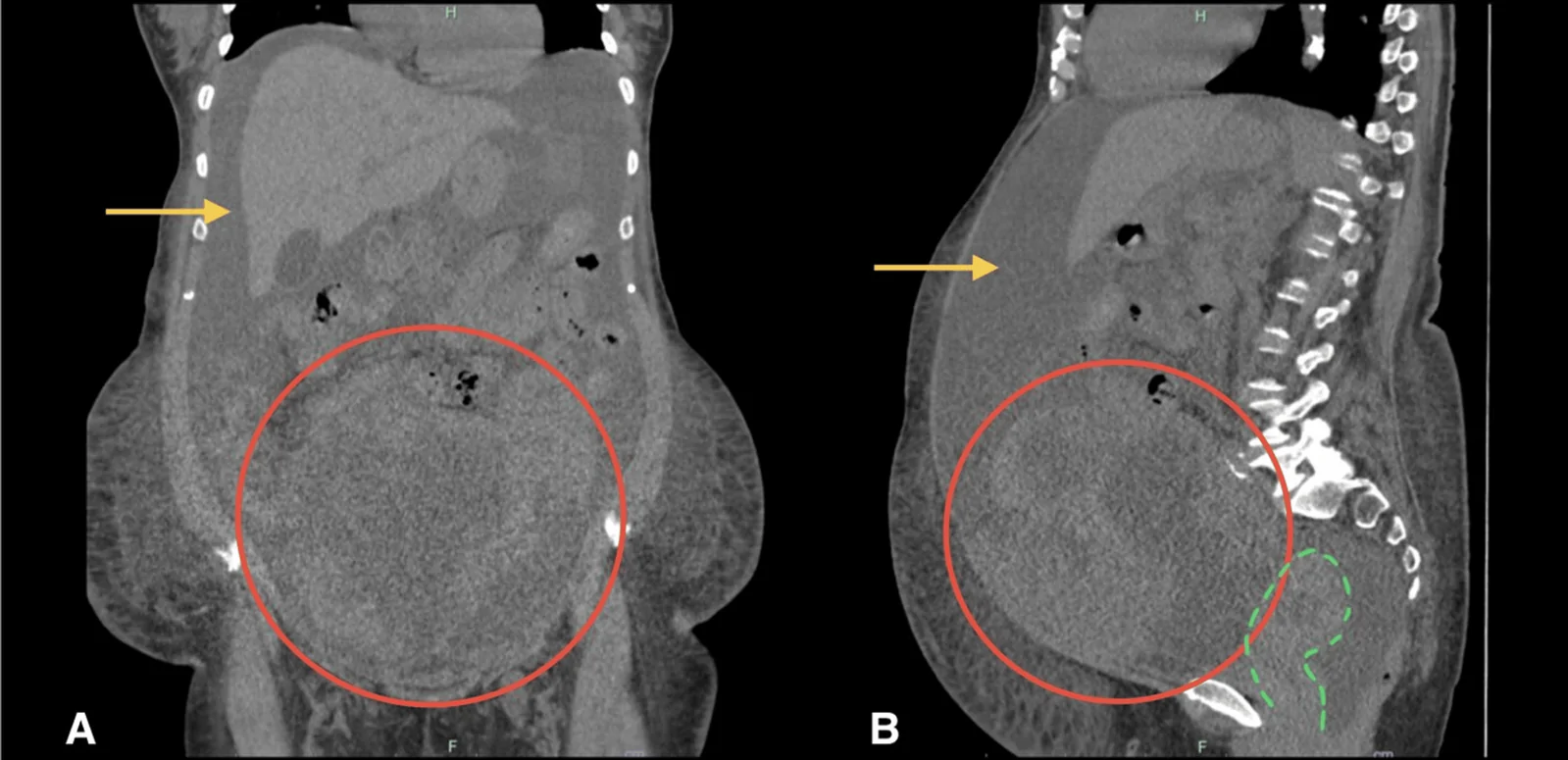
Contrast-enhanced computed tomography (CT) of the abdomen and pelvis Demonstrates a large heterogeneous adnexal mass (red circle). (A) Coronal CT image shows the lesion occupying the pelvis with associated ascitic fluid (yellow arrow); (B) Sagittal CT image confirms the extent of the mass, with ascitic fluid and the uterus (outlined by the green dashed line).

She presented with a four-month history of progressive abdominal distension, early satiety, vomiting controlled with antiemetics, bilateral lower limb edema, and mild dorsal pain. During a previous admission to a secondary hospital, contrast-enhanced computed tomography (CECT) of the abdomen and pelvis had shown abundant abdominopelvic ascites, loculated hemorrhagic collections, and a large heterogeneous solid-cystic mass measuring approximately 26 × 16 cm (Figure 1).

No suspicious lymphadenopathy or distant metastases were identified. Serum CA-125 was elevated (301 U/mL; reference range: 0-35 U/mL) at the initial patient evaluation.

**Figure 2:**
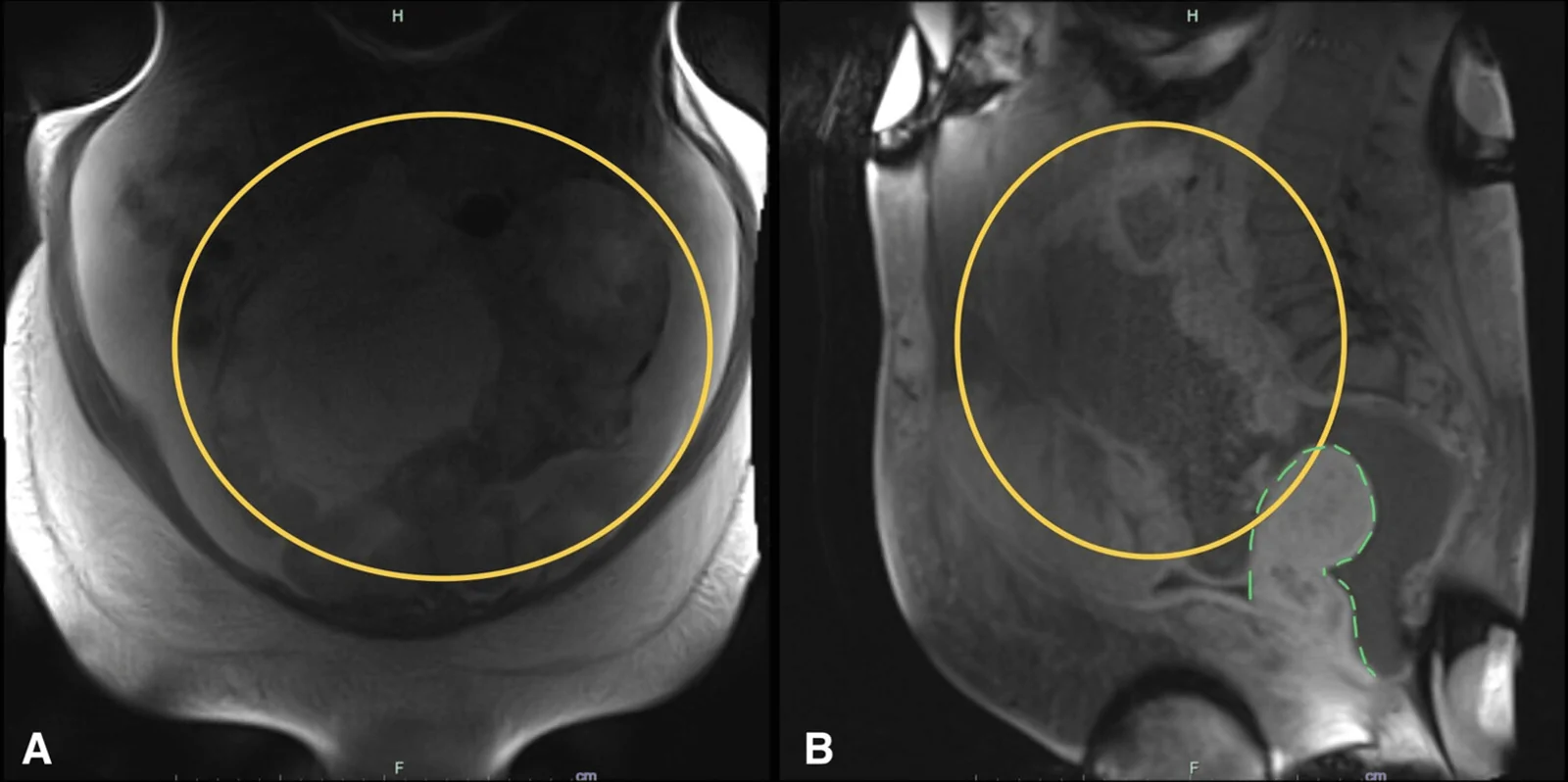
Pelvic magnetic resonance imaging (MRI) Demonstrates a large heterogeneous adnexal mass with cystic and solid components. (A) Coronal T2-weighted image shows the lesion (yellow circle) with hyperintense cystic areas and mild ascites. (B) Sagittal T1-weighted image confirms the large heterogeneous mass (yellow circle), with the uterus outlined by the green dashed line.

Pelvic magnetic resonance imaging confirmed a bulky heterogeneous adnexal mass measuring more than 25 cm, with areas suggestive of internal hemorrhage and marked mass effect on adjacent pelvic organs (Figure 2).

Imaging did not allow reliable characterization of tumor subtype. At presentation to our center, the patient had an Eastern Cooperative Oncology Group (ECOG) performance status [13] of one. Abdominal examination revealed marked distension, a large central mass extending above the umbilicus, and clinical signs of ascites.

Given the uncertain origin and histological nature of the lesion, a transvaginal ultrasound-guided biopsy was performed as a minimally invasive diagnostic procedure. Histology showed a poorly differentiated neoplasm with biphenotypic expression of epithelial and mesenchymal markers, but no definitive diagnosis could be established. A subsequent image-guided core biopsy demonstrated a spindle-cell neoplasm with smooth muscle differentiation, low proliferative activity, and minimal nuclear pleomorphism, raising the differential diagnosis of leiomyoma versus leiomyosarcoma.

Because of the atypical presentation and inconclusive pathology, an extended tumor marker and hormonal panel was obtained. CA-125 remained elevated, whereas carcinoembryonic antigen (CEA), lactate dehydrogenase (LDH), alpha-fetoprotein (AFP), and beta-human chorionic gonadotropin (β-hCG) were within normal limits. Hormonal assessment revealed markedly elevated estradiol and mildly increased testosterone levels, supporting the possibility of a hormonally active sex cord-stromal tumor.

The case was discussed at the multidisciplinary gynecologic tumor board, and surgical exploration was recommended. Repeat thoracoabdominopelvic CT showed stable ascites and peritoneal thickening, without distant metastatic disease.

Exploratory laparotomy revealed marked abdominal distension due to ascites, large bilateral adnexal masses measuring approximately 35 × 25 cm, adhesions to the anterior and lateral abdominal wall and sacral curvature, an omental mass adherent to the small and large bowel, an abdominal wall tumor involving the umbilicus, and scattered peritoneal carcinomatosis in the pouch of Douglas. Cytoreductive surgery included drainage of approximately nine liters of ascitic fluid, extensive adhesiolysis, total hysterectomy with bilateral salpingo-oophorectomy, peritonectomy of the pouch of Douglas, excision of the abdominal wall tumor including the umbilicus, and fulguration of small peritoneal implants. Complete macroscopic resection was not feasible because of unresectable adherent disease and the anticipated risk of severe morbidity. The final surgical outcome was therefore classified as R2, with residual macroscopic disease greater than 1 cm.

**Figure 3:**
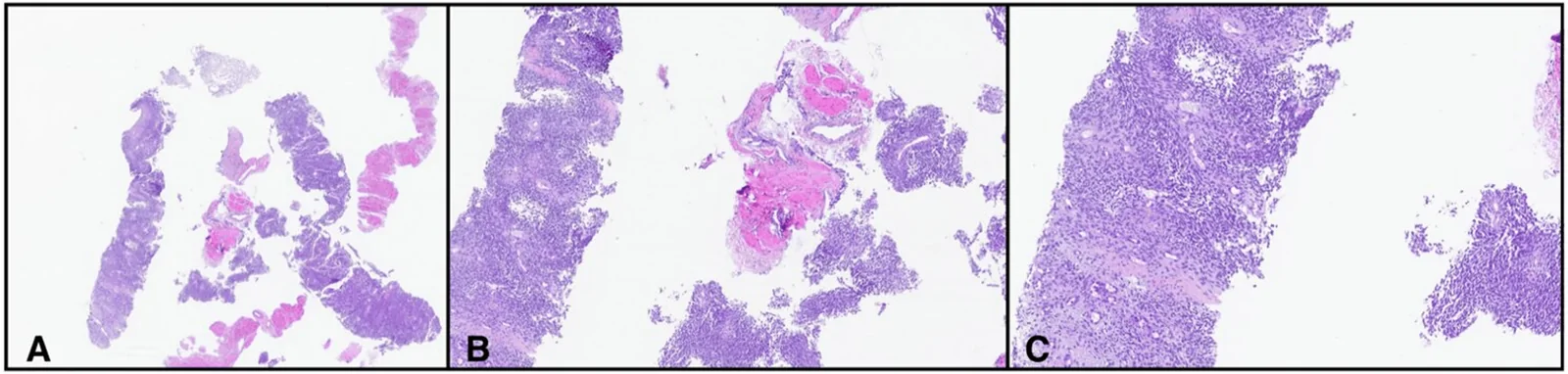
Hematoxylin and eosin-stained sections of the ovarian tumor (A) Densely cellular tumor proliferation; (B) Highly vascularized tumor areas; (C) Higher magnification demonstrating oval-to-spindle-shaped tumor cells with moderate nuclear pleomorphism.

**Figure 4:**
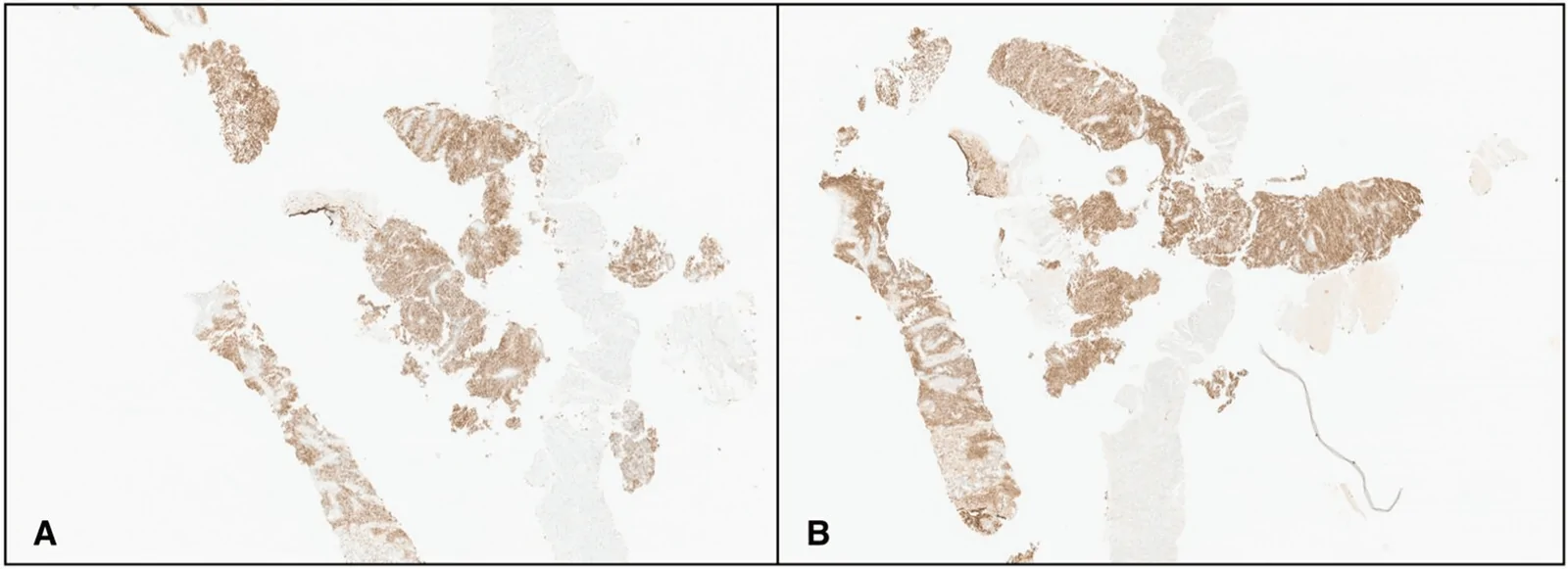
Immunohistochemical staining of the ovarian tumor (A) Diffuse cytoplasmic positivity for cytokeratin 19 (CK19); (B) Strong diffuse immunoreactivity for broad-spectrum cytokeratins anti-epithelial (AE)1/AE3, supporting epithelial differentiation.

Histopathological evaluation was complex, and the specimen was referred for expert review at a reference pathology center. The final diagnosis was an ovarian sex cord-stromal tumor most consistent with a poorly differentiated Sertoli-Leydig cell tumor with estradiol hyperproduction, staged as pT3c cN0 cM0, International Federation of Gynecology and Obstetrics (FIGO) stage IIIC. Histopathological and immunohistochemical findings are shown in Figures 3, 4. 

Molecular testing for Forkhead box L2 (FOXL2) and Dicer 1, ribonuclease III (DICER1) variants was requested, but suboptimal sample quality rendered the analysis invalid. DICER1 alterations have been reported particularly in moderately and poorly differentiated Sertoli-Leydig cell tumors, supporting their relevance in the molecular characterization of this entity.

In view of advanced disease, residual tumor, and the risk of clinical deterioration, systemic therapy was proposed. Considering the patient’s age, comorbidities, and performance status, carboplatin-paclitaxel was selected instead of a cisplatin-based regimen. She received carboplatin area under the curve (AUC) four and paclitaxel at 85% of the standard dose, aiming to balance disease control and tolerability. Supportive care included analgesia, antiemetics, gastric protection, nutritional counseling, and anticoagulation.

The patient completed nine cycles of carboplatin-paclitaxel. Postoperative CT performed approximately three months after surgery showed a large loculated cystic mass in the lesser sac, persistent moderate ascites, and diffuse peritoneal nodularity consistent with peritoneal carcinomatosis, without distant metastases. Tumor markers were normal, but serum estradiol remained markedly elevated.

Approximately one year after surgery, CT demonstrated persistent large-volume ascites, a complex loculated cystic lesion, and worsening peritoneal carcinomatosis, including implants adherent to the abdominal wall and paracolic gutter. Biopsy of a new abdominal wall lesion confirmed recurrent poorly differentiated Sertoli-Leydig cell tumor involving the linea alba. Further image-guided biopsy of intra-abdominal lesions was considered to better characterize progressive disease, but it was not feasible because of extensive malignant ascites and the absence of a safely accessible solid target.

The patient required repeated admissions for symptom control, including large-volume paracenteses of hemorrhagic ascites. Despite supportive measures, her condition deteriorated, with progressive abdominal distension, early satiety, abdominal colic, and wheelchair dependence. Her performance status declined to ECOG two, and laboratory evaluation showed anemia, renal impairment, electrolyte disturbances, and elevated LDH.

After discussion with the patient and family, weekly paclitaxel was proposed as palliative second-line therapy. Best supportive care alone was also discussed as an appropriate option, but the patient and relatives preferred to proceed with chemotherapy. Her clinical condition rapidly deteriorated after treatment initiation, and she died approximately one week later.

## Discussion

This case illustrates the diagnostic and therapeutic complexity of advanced poorly differentiated ovarian Sertoli-Leydig cell tumors in elderly patients. Although most Sertoli-Leydig cell tumors are diagnosed at an early stage and have favorable outcomes, poorly differentiated tumors are associated with a higher risk of recurrence, extraovarian spread, and reduced survival [1-5]. Presentation in older women is uncommon and may be associated with non-specific symptoms, larger tumor burden, and delayed diagnosis [1,2]. Presentation in older women and outcomes in advanced disease have also been described in institutional series and retrospective cohorts [14].

The diagnostic pathway in this case was particularly challenging. Initial biopsies showed overlapping epithelial and mesenchymal features, raising the possibility of alternative diagnoses, including mesenchymal tumors of uterine or adnexal origin. This reflects a recognized pitfall of poorly differentiated sex cord-stromal tumors, which may display heterogeneous morphology and a broad immunohistochemical profile [3,5]. In such cases, expert pathology review is essential to avoid misclassification and to guide appropriate management [3,5].

Hormonal evaluation was a useful diagnostic clue. Markedly elevated estradiol levels, together with mildly increased testosterone, supported the diagnosis of a hormonally active sex cord-stromal tumor. Estrogen-producing Sertoli-Leydig cell tumors are uncommon but have been reported, and endocrine evaluation may provide additional diagnostic information in atypical cases [6,7]. Persistent postoperative estradiol elevation was observed throughout follow-up. This finding was most consistent with active residual disease, although hormonal markers should be interpreted in conjunction with clinical and radiological findings, as their relationship with tumor burden may be variable.

Surgery remains the cornerstone of treatment for Sertoli-Leydig cell tumors, particularly in advanced or poorly differentiated disease [8,9]. Advanced stage, poor differentiation, and residual macroscopic disease were all adverse prognostic factors [4,9]. Complete macroscopic cytoreduction (R0/CC-0) remains one of the most important prognostic factors in ovarian malignancies and should be pursued whenever safely feasible. In the present case, complete resection was precluded by extensive unresectable disease and the anticipated risk of severe surgical morbidity, highlighting the adverse prognostic implications of residual disease.

Molecular characterization may also be relevant. DICER1 mutations have been frequently described in moderately and poorly differentiated Sertoli-Leydig cell tumors, whereas FOXL2 testing may assist in the differential diagnosis with other ovarian sex cord-stromal tumors [3,10,11]. In the present case, molecular testing was attempted, but suboptimal sample quality prevented completion of the analysis.

The optimal systemic treatment for advanced ovarian sex cord-stromal tumors remains uncertain. Bleomycin, etoposide, and cisplatin have historically been used, particularly in younger patients, but bleomycin- and cisplatin-related toxicities may limit its use in elderly or comorbid patients [12]. Carboplatin-paclitaxel may therefore represent a reasonable alternative when the goal is to achieve disease control with a more favorable tolerability profile [12,13]. In this patient, carboplatin-paclitaxel allowed the completion of nine cycles, but the disease ultimately progressed.

This case also underscores the need for early integration of palliative care in aggressive rare ovarian tumors. When evidence-based options are limited and prognosis is poor, treatment decisions should incorporate patient preference, symptom burden, frailty, expected toxicity, and quality of life [8,9,12,15].

## Conclusions

In conclusion, poorly differentiated ovarian Sertoli-Leydig cell tumors are rare neoplasms that may present with marked diagnostic ambiguity and aggressive clinical behavior, particularly in elderly patients. In this case, inconclusive biopsies, overlapping histopathological features, and atypical endocrine findings complicated the diagnostic process, highlighting the importance of expert pathological review and multidisciplinary evaluation. Residual macroscopic disease and progression despite systemic therapy illustrate the therapeutic challenges encountered in advanced-stage disease, particularly in frail or comorbid patients in whom treatment tolerability must be carefully balanced against potential benefit. This case underscores the need for individualized treatment planning and early integration of supportive and palliative care strategies in patients with aggressive rare ovarian tumors.

## References

[1] Xu Q, Zou Y, Zhang XF (2018). Sertoli-Leydig cell tumors of ovary: a case series. Medicine (Baltimore).

[2] Wang G, Zhang R, Li C, Chen A (2021). Characteristics and outcomes analysis of ovarian Sertoli-Leydig cell tumors (SLCTs): analysis of 15 patients. J Ovarian Res.

[3] Němejcová K, Hájková N, Krkavcová E (2025). A molecular and immunohistochemical study of 37 cases of ovarian Sertoli-Leydig cell tumor. Virchows Arch.

[4] Nelson AT, Harris AK, Watson D (2024). Outcomes in ovarian Sertoli-Leydig cell tumor: a report from the International Pleuropulmonary Blastoma/DICER1 and Ovarian and Testicular Stromal Tumor registries. Gynecol Oncol.

[5] (2026). Female Genital Tumours. WHO Classification of Tumours, 5th Edition, Volume 4. https://publications.iarc.who.int/Book-And-Report-Series/Who-Classification-Of-Tumours/Female-Genital-Tumours-2020.

[6] Tsuzuki Y, Kikuchi I, Nojima M, Yoshida K, Hashizume A, Tomita S (2017). A case report: ovarian Sertoli-Leydig cell tumor with hyperestrogenism and endometrial hyperplasia in a postmenopausal woman. Jpn Clin Med.

[7] Chong YY, Wong JL, Ang XH, Lim YL (2024). Sertoli-Leydig cell tumor as a cause of elevated 17-OH progesterone. JCEM Case Rep.

[8] Ray-Coquard I, Morice P, Lorusso D (2018). Non-epithelial ovarian cancer: ESMO Clinical Practice Guidelines for diagnosis, treatment and follow-up. Ann Oncol. 2018.

[9] Calzas Rodríguez J, Carmen Juarez Morales MD, Casero MA (2016). Death by bleomycin pulmonary toxicity in ovarian dysgerminoma with pathologic complete response to chemotherapy. A case report. Respir Med Case Rep.

[10] Kato N, Kusumi T, Kamataki A, Tsunoda R, Fukase M, Kurose A (2017). DICER1 hotspot mutations in ovarian Sertoli-Leydig cell tumors: a potential association with androgenic effects. Hum Pathol.

[11] Yuan Z, Huo X, Jiang D (2020). Clinical characteristics and mutation analyses of ovarian Sertoli-Leydig cell tumors. Oncologist.

[12] Brink GJ, Groeneweg JW, Hooft L, Zweemer RP, Witteveen PO (2022). Response to systemic therapies in ovarian adult granulosa cell tumors: a literature review. Cancers (Basel).

[13] Oken MM, Creech RH, Tormey DC, Horton J, Davis TE, McFadden ET, Carbone PP (1982). Toxicity and response criteria of the Eastern Cooperative Oncology Group. Am J Clin Oncol.

[14] Bhat RA, Lim YK, Chia YN, Yam KL (2013). Sertoli-Leydig cell tumor of the ovary: analysis of a single institution database. J Obstet Gynaecol Res.

[15] Castro BG, Souza CP, Andrade CE, Vieira MA, Andrade DA, Reis RD (2019). Ovarian Sertoli-Leydig cell tumors: epidemiological, clinical and prognostic factors. Rev Bras Ginecol Obstet.

